# CD4^+^ but not CD8^+^ T cells revert the impaired emotional behavior of immunocompromised *RAG-1*-deficient mice

**DOI:** 10.1038/tp.2013.54

**Published:** 2013-07-09

**Authors:** L Rattazzi, G Piras, M Ono, R Deacon, C M Pariante, F D'Acquisto

**Affiliations:** 1 Centre for Biochemical Pharmacology, The William Harvey Research Institute, Barts and the London School of Medicine and Dentistry, Queen Mary University of London, London, UK; 2 Institute of Child Health, University College London, London, UK; 3 Department of Experimental Psychology, University of Oxford, Oxford, UK; 4 Department of Psychological Medicine, Institute of Psychiatry, King's College London, London, UK

**Keywords:** CD4 T cells, CD8 T cells, immunodeficient mice, OT-I and OT-II transgenic mice, *RAG* knockout mice

## Abstract

An imbalanced immune system has long been known to influence a variety of mood disorders including anxiety, obsessive-compulsive disorders and depression. In this study, we sought to model the impact of an immunocompromised state on these emotional behaviors using *RAG-1^−/−^* mice, which lack T and B cells. We also investigated the relative contribution of CD4^+^ or CD8^+^ T cells to these manifestations using *RAG-1^−/−^*/OT-II and *RAG-1^−/−^*/OT-I transgenic mice, respectively. Our results show that *RAG-1^−/−^* mice present a significant increase in digging and marble-burying activities compared with wild-type mice. Surprisingly, these anxiety-like behaviors were significantly reverted in *RAG-1^−/−^*/OT-II but not *RAG-1^−/−^*/OT-I transgenic mice. Immunodepletion experiments with anti-CD4 or anti-CD8 in C57/BL6 mice or repopulation studies in *RAG-1^−/−^* mice did not reproduce these findings. Microarray analysis of the brain of *RAG-1^−/−^* and *RAG-1^−/−^*/OT-II mice revealed a significantly different gene fingerprint, with the latter being more similar to wild-type mice than the former. Further analysis revealed nine main signaling pathways as being significantly modulated in *RAG-1^−/−^* compared with wild-type mice. Taken together, these results suggest that life-long rather than transient immunodeficient conditions influence the emotional behaviors in mice. Most interestingly, these effects seem to correlate with a specific absence of CD4^+^ rather than CD8^+^ T cells. Validation of these findings in man might provide new clues on the mechanism by which early life immune modulation might impact mood response in adults and provide a further link between immune and emotional well-being.

## Introduction

A correlation between mental diseases and immune dysfunction has been reported and debated in the literature since the late 1980s.^1^ Indeed, direct and indirect evidences in both human and animal experimental systems indicate that changes in the immune repertoire significantly influence cognitive functions^2, 3^ and neurodegeneration.^4, 5, 6^ More recent studies also suggest that a healthy immune system is a prerequisite for a balanced and functional emotional response.^7, 8, 9, 10^

The link between emotion and immunity has been documented in a variety of studies addressing psychosocial changes in patients treated with immunosuppressive drugs. Cyclosporine, a drug widely used in organ transplantation, has been shown to induce a range of neuropsychological problems ranging from depression to anxiety.^11, 12, 13, 14, 15^ Similarly, other studies described psychological side effects like anxiety, depression and obsessive-compulsive disorders in patients treated with a variety of structurally unrelated immunosuppressant including methotrexate,^16^ azathioprine,^17^ and chemotherapy.^18^

The recombination-activating gene *RAG-1* encodes proteins necessary for immunoglobulin and T-cell receptor gene recombination. *RAG-1*-deficient mice have small lymphoid organs that do not contain mature B and T lymphocytes.^19^ Seminal work on *RAG-1^−/−^* mice by Cushman and co-workers^20^ reported an increased locomotor activity, reduced levels of fearfulness and no differences in spatial learning and memory. However, given the shared expression of the *RAG-1* protein by lymphocytes and central nervous system tissues, the authors concluded their seminal paper stating: ‘Whether these changes are due to the loss of *RAG-1* gene expression in the brain, the result of the absence of the *RAG-1* gene in the immune system or some combination of both effects remains to be determined in future research'.

To address this question, McGowan *et al.*^21^ used a very elegant approach to assess the function of *RAG-1* in the central nervous system and dissect it from the lack of T and B cells in the periphery.^21^ The authors compared the behavior of *RAG-1* to*RAG*-2-deficient mice and found an impaired social recognition memory in the first but not the latter. Because both lines are immunodeficient and *RAG*-2 is not expressed in the brain, the authors claimed a specific function of *RAG-1* in controlling memory formation.

In this study, we expanded on these notions and explored first if the immunodeficient state of *RAG-1^−/−^* mice had any influence on their emotional behavior and second the specific contribution of CD4^+^ or CD8^+^ T cells to these changes using *RAG-1^−/−^*/OT-II and the *RAG-1^−/−^*/OT-I transgenic lines, respectively. Second, we explored possible changes in mood-modifying circulating factors or gross brain structural differences in these mice. The results obtained suggest that CD4^+^ but not CD8^+^ T cells are capable of partially reverting anxiety-like behavior and lack of self-care characteristic of immunodeficient mice. Interestingly, these behavioral changes were mirrored in specific brain gene fingerprint. These unexpected new findings might provide a mechanistic explanation for the increased emotional distress observed in patients suffering from a wide variety of immune disorders.

## Materials and methods

### Mice

We used 5-week-old male mice for all the experiments. Mice were housed in groups of six per cage under specific pathogen-free conditions and with free access to food and water. Mice were housed for at least 7 days before testing. Wild-type (WT) C57BL/6 mice purchased from Charles River (Manston, UK) or *RAG-1^+/+^* littermate were used as control. B6.SJL-*Ptprc*^*a*^ *Pepc*^*b*^/BoyJ-Tg(TcraTcrb)1100Mjb/J-B6.129S7-*Rag1*^*tm1Mom*^ (*RAG^−/−^*/OT-I) and B6.SJL-*Ptprc*^*a*^ *Pepc*^*b*^/BoyJ-Tg(TcraTcrb)425Cbn/J-B6.129S7-*Rag1*^*tm1Mom*^ (*RAG^−/−^*/OT-II) mice were kindly provided by Professor Hans Stauss (University College London, London, UK) and bred in our animal facility. Apart from the nest construction test, all experiments were performed during the light phase of the light–dark cycle, and no more than two tests per day were performed. All tests were conducted in a blinded manner and according to the UK Animals (Scientific Procedures) Act, 1986.

### Flow cytometric analysis

Thymocytes and lymphocytes were stained in 100 μl of fluorescence-activated cell sorting buffer (phosphate-buffered saline containing 5% fetal calf serum and 0.02% of NaN_2_). The antibodies used were anti-CD3 phycoerythrin (clone 145-2C11), anti-CD4 fluorescein isothiocyanate (clone GK 1.5), anti-CD8 Cy5 (clone 53-6.7) (all from eBioscience, San Diego, CA, USA). Cells were labeled with the appropriate concentration of conjugated antibodies for 1 h at 4 °C as described previously.^22^ After labeling, cells were washed and analyzed. In all experiments, stained cells were acquired with FACScalibur flow cytometer and analyzed using the FlowJo^TM^ software (Tree Star, Ashland, OR, USA, Oregon Corporation).

### *In vivo* T-cell depletion

Male C57/BL6 mice (6 weeks old) received an intraperitoneal injection of anti-CD4 (250 μg; clone GK1.5; BioLegend) or anti-CD8 (250 μg; clone 53-6.7; BioLegend, San Diego, CA, USA) or immunoglobulin G control. T-cell immunodepletion was verified by staining peripheral blood mononuclear cells at different time points (Day 2, Day 5 and Day 7) after the treatment. Briefly, blood samples were collected by intracardiac puncture in syringes containing sodium citrate 3.2% (w v^−1^). The cells were centrifuged to pellet at 300 *g* and resuspended in fluorescence-activated cell sorting buffer containing 1:500 Fc blocking (anti-mouse CD16/32) and stained with anti-CD4 or anti-CD8. Red blood cells were lysed with RBC Lysis Buffer according to the manufacturer's instruction (eBioscience).

### *RAG1^−/−^* repopulation studies

Purified CD4 or CD8 T cells were obtained from male C57/BL6 mice (6 weeks old) by negative selection following the manufacturer's instructions (Dynabeads® Untouched™ Mouse CD8 Cells and Dynabeads® Untouched™ Mouse CD4 Cells; Invitrogen, Invitrogen Life Technologies Ltd, Paisley, UK). Purity was tested by fluorescence-activated cell sorter and was >98%. Cells were resuspended in phosphate-buffered saline (2 × 10^6^/300 l) and transferred into male *RAG1^−/−^* mice (6 weeks old) by intraperitoneal injection.

### Digging and marble-burying tests

Marble-burying and digging tests were carried out as described previously^23^ with some modifications. Briefly, mice were individually placed in a clear plastic box (14 cm × 10 cm × 11 cm) filled with approximately 5-cm-deep wood chip bedding lightly pressed to give a flat surface. The same bedding substrate was used for all the mice and flattened after each test. Fifteen glass marbles were placed on the surface in five rows of three marbles each. The latency to start digging, the number of digging bouts and the number of buried marbles (to 2/3 their depth) were recorded during the 15-min test. Two trials were performed, the second trial taking place 24 h after the first trial.

### Open field activity test

The open filed test was performed as described previously with some modifications.^24^ The open field consisted of a white PVC arena (50 cm × 30 cm) divided into 10 cm × 10 cm squares (*n*=15). Mice were brought into the experimental room 15 min before testing. Each mouse was placed in one of the corner squares facing the wall, observed and recorded for 5 min. The total number of squares crossed, latency to the first rear and the total number of rears were recorded. After each test, the arena was cleaned with water to attenuate and homogenize olfactory traces. Two trials were performed, the second trial taking place 24 h after completion of the first trial.

### Nest construction test

The nest construction test was performed as described previously.^25^ Mice were transferred into individual cages 1 h before the dark phase (1700 hours) and individually housed overnight. The results were assessed the next morning. Food, water and wood chip bedding were provided. No other environmental enrichment was added. One 2–3 g, 5 cm × 5 cm pressed cotton square (nestlet; Ancare, Ancare Bellmore, NY, USA) was placed in each cage. The weight of nesting material shredded was calculated by weighing the nestlet before and after the overnight test. The quality of the nest was evaluated on a five-point scale as detailed in Deacon.^25^

### Plasma corticosterone and cytokine measurement

Blood was collected by intracardiac puncture performed under anesthesia. Serum was obtained from the clotted blood by centrifugation (8000 r.p.m., 5 min) and stored at −80 °C before the assay. Corticosterone concentrations were measured in diluted (1:32) plasma by EIA assay following the manufacturer's instructions (Enzo Life Sciences, Exeter, UK). Cytokine levels in the same samples were measured (dil. 1:100) using mouse T-helper type 1 (Th1)/Th2/Th17/Th22 16plex Kit Flowcytomix and according to the manufacturer's instructions (eBioscience).

### Histology

Brains were collected either before or at the end of experiments and fixed in 4% paraformaldehyde for 72 h. Thereafter, tissues were sectioned on a sagittal or coronal plane and embedded in paraffin by our in-house histology facility. Sections (5 μm) were deparaffinized and stained with hematoxylin and eosin. In all cases, a minimum of three sections per animal was evaluated. Phase-contrast digital images were taken using the Image Image-Pro (Media Cybernetics, Rockville, MD, USA) analysis software package.

### Microarray analysis

Total RNA was extracted from brains of WT (*n*=3), *RAG^−/−^* (*n*=2) and OT-II/*RAG^−/−^* (*n*=2) mice using RNeasy® Microarray Tissue Mini Kit (Qiagen®, West Sussex, UK). Total RNA was hybridized to Affymetrix Mouse Gene 1.0 ST array chips at UCL Genomics (London, UK) with standard Affymetrix protocols, using GeneChip Fluidics Station 450, and scanned using the Affymetrix GeneChip Scanner (Affymetrix, Santa Clara, CA, USA). Data were normalized by *rma* of the Bioconductor package, *affy*. Differentially expressed genes were identified by the Bioconductor package, *limma*, considering the false discovery rate (adjusted *P*-value <0.05). The gene and sample scoring system was made by canonical correspondence analysis. Canonical correspondence analysis is a variant of correspondence analysis, where the main data are linearly regressed onto explanatory variables (environmental variables), and subsequently the regressed data are analyzed by correspondence analysis. In this study, we regressed the whole data set onto an explanatory variable, which was defined as the difference between ‘average' WT and ‘average' *RAG-1^−/−^*. Detailed methodology is described elsewhere.^26^ Signaling pathway impact analysis was performed using the Bioconductor package, *SPIA*, by comparing WT and *RAG-1^−/−^*.

### Real-time polymerase chain reaction

Total RNA was extracted from whole brains (*n*=6 for each mouse line) with RNeasy® Microarray Tissue Mini Kit (Qiagen®) according to the manufacturer's protocol and reverse transcribed using 2 μg oligo(dT)15 primer, 10 U AMV reverse transcriptase, 40 U RNase inhibitor (all from Promega Corporation, Madison, WI, USA) and 1.25 mℳ each dNTP (Bioline, London, UK) for 45 min at 42 °C. Real-time polymerase chain reaction was carried out by using ABsoluteTM QPCR ROX Mix (Thermo Scientific, Epsom, UK) and fluorescent QuantiTect primers. Cycling conditions were set according to the manufacturer's instructions. Sequence-specific fluorescent signal was detected by 7900HT Fast Real-Time PCR System (Applied Biosystems, Warrington, Cheshire, UK). mRNA data were normalized relative to glyceraldehyde 3-phosphate dehydrogenase and then used to calculate expression levels. We used the comparative Ct method to measure the gene transcription in samples. The results are expressed as relative units based on calculation of 2^−ΔΔCt^, which gives the relative amount of gene normalized to endogenous control (glyceraldehyde 3-phosphate dehydrogenase) and to the sample with the lowest expression set as 1.

### Data analysis

Initially, we determined if the data distribution was parametric. Pairwise comparisons were made by *t*-test and the results expressed as mean±s.e.m. For non-parametric data, the Mann–Whitney *U*-test was applied, and results were expressed as medians (interquartile range).

## Results

### Immune repertoire of *RAG-1^−/−^*, *RAG-1^−/−^*/OT-I and *RAG-1^−/−^*/OT-II

**Figure 1:**
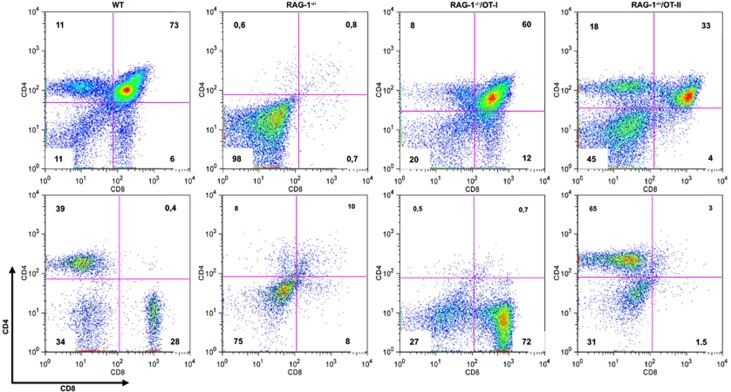
Immune repertoire of *RAG-1^−/−^*, *RAG-1^−/−^*/OT-I and *RAG-1^−/−^*/OT-II mice. Thymocytes and lymphocytes from male wild-type (WT), *RAG-1^−/−^*, *RAG-1^−/−^*/OT-I and *RAG-1^−/−^*/OT-II mice were analyzed for CD4 and CD8 expression. The dot plots show the T-cell profiles of *RAG-1^−/−^*, *RAG-1^−/−^*/OT-I and *RAG-1^−/−^*/OT-II mice in the thymus (upper panel) and in the periphery (lower panel) compared with WT C57BL/6 control mice. The percentage of the cells in each quadrant is given.

Crossing OT-I and OT-II TCR transgenic mice onto *RAG-1^−/−^* background generates mice with a single population of mature CD8^+^ or CD4^+^ T cells. Figure 1 shows a typical immature and mature T-cell profiles of *RAG-1^−/−^*, *RAG-1^−/−^*/OT-I and *RAG-1^−/−^*/OT-II compared with WT C57BL/6 control mice. Control mice show a typical profile with a 1:2 ratio of CD8^+^ and CD4^+^ single-positive T cells in the thymus as well as in the periphery (Figure 1, first top and bottom panels, respectively). As expected, the majority of *RAG-1^−/−^* thymocytes are CD4 and CD8 double-negative cells and have no mature CD4 or CD8 single-positive T cells in the periphery (Figure 1, second top and bottom panels, respectively).^19^ The presence of OT-I and OT-II TCR transgene overcomes the block at the stage of double-negative 3 (DN3) of *RAG-1^−/−^* thymocytes and allows the generation of a peripheral T-cell immune repertoire constituted by 72% of CD8^+^ in *RAG-1^–/–^*/OT-I and 65% of CD4^+^ T cells *RAG-1^–/–^*/OT-II mice (Figure 1, third top and bottom panels).^27^

### Increased digging and marble-burying behavior of *RAG-1^−/−^* mice

**Figure 2:**
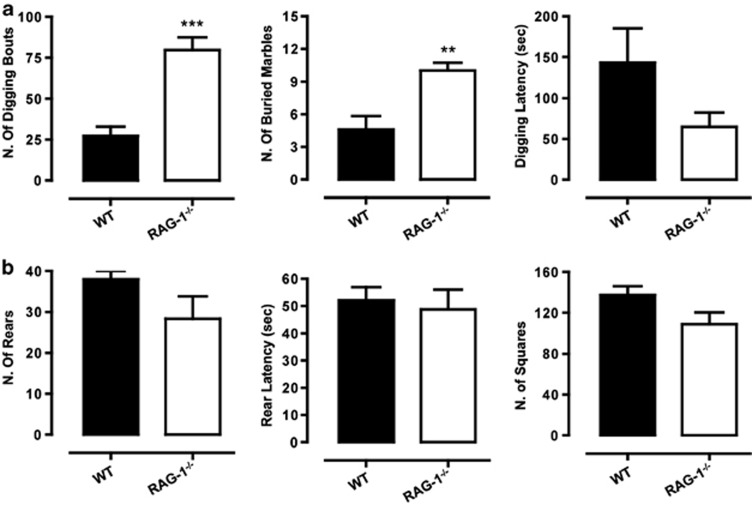
Increased digging and marble-burying behavior of *RAG-1^−/−^* mice (upper panel). The bar graphs in **a** shows the total number of digging bouts, buried marbles and the latency to dig (expressed in seconds) measured during the 15min marble-burying test. The bar graphs in **b** show the total number of rears, the latency to rear (expressed in seconds) and the total number of squares crossed assessed during the 5-min open field test. Values are expressed as mean±s.e.m. of six mice and are representative of *n*=3–4 separate experiments. **P<0.01 and ***P<0.001 indicate significant values compared with wild-type (WT) C57BL/6 control mice (Mann–Whitney *U*-test).

We first investigated differences in anxiety- and obsessive-compulsive-like behavior in WT and *RAG-1^−/−^* mice using the digging and marble-burying tests. As shown in Figure 2a, *RAG-1^−/−^* mice presented a significant increase (two- to threefold) in the number of digging bouts and buried marble compared with WT mice. The latency to dig was higher in control mice compared with *RAG-1^−/−^* mice, although the difference was not significant.

To further corroborate the anxiety-like behavior and simultaneously rule out possible intrinsic impairment in locomotor activity, we used the open field activity test. Here, we considered a number of parameters including exploration (number of rears and latency) and locomotor activity (number of squares crossed). Although *RAG-1^−/−^* mice showed a slight reduction compared with the mice in all the parameters observed, these differences were not statistically significant (Figure 2b).

### CD4^+^ but not CD8^+^ T cells revert the increased digging and marble-burying behavior of *RAG-1^−/−^* mice

**Figure 3:**
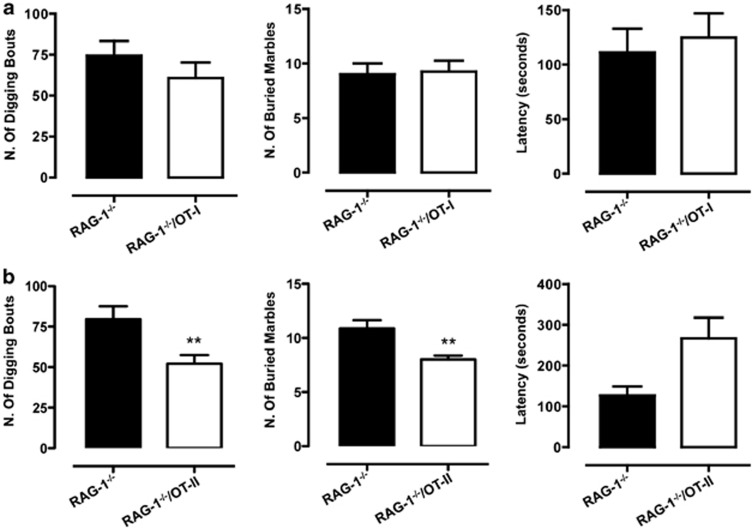
CD4^+^ but not CD8^+^ T cells revert the increased digging and marble-burying behavior of *RAG-1^−/−^* mice. The bar graphs show the total number of digging bouts, buried marbles and the latency to dig (expressed in seconds) in *RAG-1^−/−^*/OT-I (**a**) or *RAG-1^−/−^*/OT-II (**b**) compared with *RAG-1^−/−^* during the 15-min marble-burying test. Values are expressed as mean±s.e.m. of six mice and are representative of *n*=3–4 separate experiments. **P<0.01 indicates significant values compared with *RAG-1^−/−^* mice (Mann–Whitney *U*-test).

We next tested the hypothesis that the presence of CD4^+^ or CD8^+^ T cells might influence the heightened digging and marble-burying behavior of the *RAG-1^−/−^* mice. *RAG-1^–/–^*/OT-I showed no difference in either number of bouts, buried marbles or latency compared with *RAG-1^−/−^* mice (Figure 3a). In contrast, *RAG-1^–/–^*/OT-II behaved differently from *RAG-1^−/−^* mice, showing a significant reduction in the number of bouts (about 25%) and buried marbles (about 20%), and almost doubled latency time (*P*<0.05; Figure 3b).

**Figure 4:**
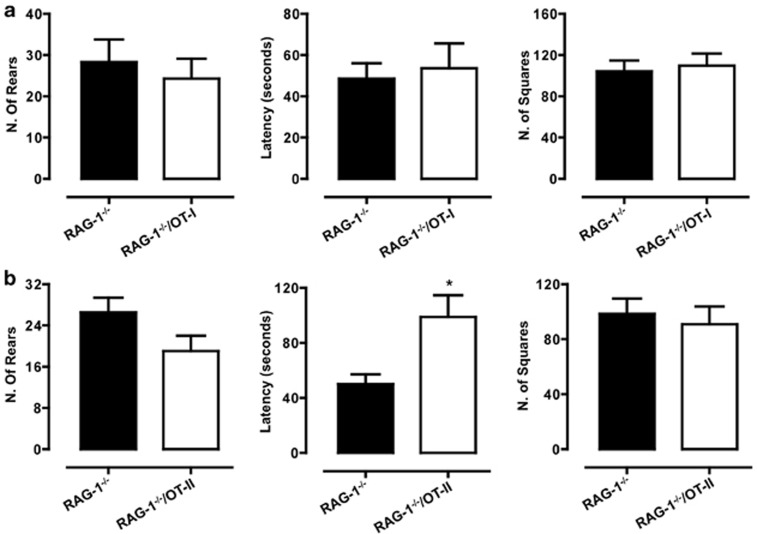
No differences in the open field activity between *RAG-1^−/−^*/OT-I, *RAG-1^−/−^*/OT-II and *RAG-1^−/−^*. The bar graphs show the total number of rears, the latency to rear (expressed in seconds) and squares crossed in *RAG-1^−/−^*/OT-I (**a**) or *RAG-1^−/−^*/OT-II (**b**) compared with *RAG-1^−/−^* during the 5-min open field test. Values are expressed as mean±s.e.m. of six mice and are representative of *n*=3–4 separate experiments. *P<0.05 indicates significant values compared with *RAG-1^−/−^* mice (Mann–Whitney *U*-test).

**Figure 5:**
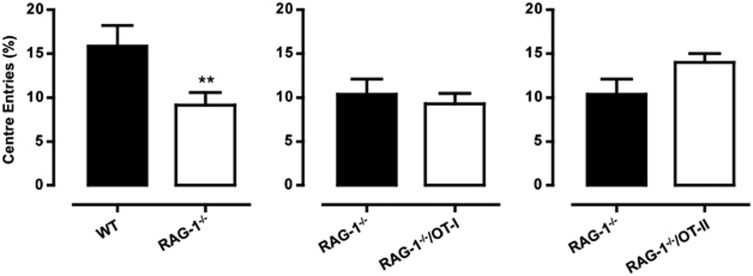
CD4^+^ but not CD8^+^ T cells might revert the decreased number of center entries showed by *RAG-1^−/−^* mice. The bar graphs show the comparison of the entries into the center between *RAG-1^−/−^* and wild-type mice (left panel), *RAG-1^−/−^* and *RAG^−/−^*/OT-I (middle panel) and *RAG^−/−^* and *RAG^−/−^*/OT-II (right panel) during the 5-min open field test. Values are expressed as mean±s.e.m. of six mice and are representative of *n*=3–4 separate experiments. **P<0.01 indicates significant values compared with wild-type C57BL/6 control mice (Mann–Whitney *U*-test).

With the open field test, we observed no difference in the behavior of *RAG-1^–/–^*/OT-I and *RAG-1^–/–^* (Figure 4a). Similarly, *RAG-1^–/–^*/OT-II showed no significant difference in either the number of rears or of squares crossed as compared with *RAG-1^−/−^*, except for an increase in the latency, which this time reached a statistical significance (Figure 4b). When we compared the number of center entries (considered an anxiety-like behavior parameter) in the three lines, we observed a significant reduction in *RAG-1^−/−^* mice compared with WT mice. However, this difference was not significantly reverted in either *RAG-1^−/−^*/OT-I or *RAG-1^−/−^*/OT-II (Figure 5).

### Transient depletion of CD4^+^ or CD8^+^ T cells does not affect the emotional behavior of C57/BL6 mice

**Figure 6:**
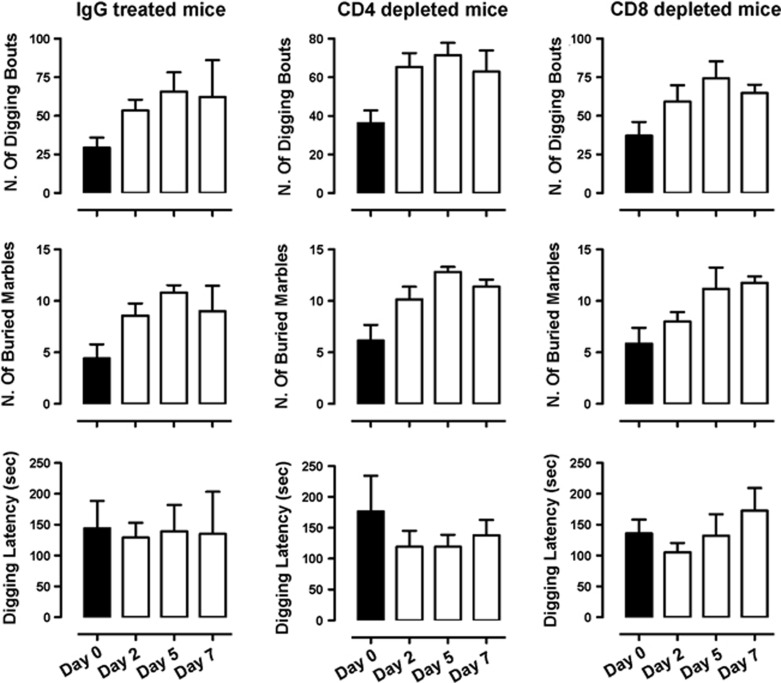
Depletion of CD4 or CD8 T cells does not induce anxiety-like behavior in C57/BL6 mice. C57/BL6 mice received an intraperitoneal injection of anti-CD4 (250 μg), anti-CD8- (250 μg) depleting antibodies or IgG control, and then tested in the digging and marble-burying test. The bar graphs show the total number of digging bouts, buried marbles and the latency to dig (expressed in seconds) in mice treated as indicated and assessed before the treatment (day 0) or after 2, 5 and 7 days (day 2, day 5 and day 7, respectively). Values are expressed as mean±s.e.m. of 6–8 mice.

We next wondered if we could reproduce the results obtained with *RAG-1^–/–^*/OT-I and *RAG-1^–/–^*/OT-II using anti-CD4- or anti-CD8-depleting antibodies in C57/BL6 mice. As shown in Figure 6, neither anti-CD4 nor anti-CD8 antibodies significantly modified the digging, or marble-burying activities or the latency to digging (top, middle and bottom panels, respectively) compared with control IgG-treated mice at the indicated times.

**Figure 7:**
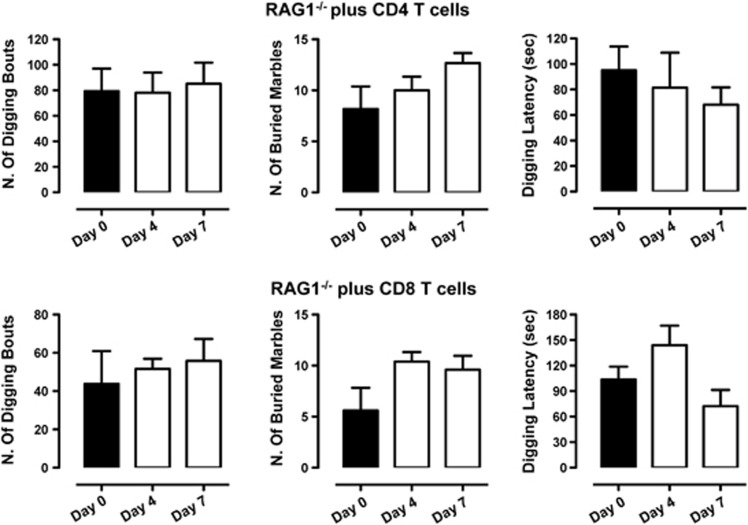
Repopulation of *RAG1^−/−^* mice with CD4 or CD8 T cells does not affect their anxiety-like behavior. *RAG1^−/−^* mice received an intraperitoneal injection of purified CD4 (2 × 10^6^) or CD8 (2 × 10^6^) T cells and then tested in the digging and marble-burying test. The bar graphs show the total number of digging bouts, buried marbles and the latency to dig (expressed in seconds) in mice assessed before the cell transfer (day 0) or after 4 and 7 days (day 4 and day 7, respectively). Values are expressed as mean±s.e.m. of 6–8 mice.

To further confirm these results, we sought to investigate if the repopulation of *RAG-1^−/−^* with T cells would rescue the increased anxiety observed in these mice. Consistent with previous results, reconstitution of *RAG-1^−/−^* mice with purified CD4 or CD8 T cells did not affect the increase in the number of digging bouts compared with vehicle phosphate-buffered saline-injected mice (Figure 7).

### CD4^+^ T cells revert the impaired nest construction of *RAG-1^−/−^* mice

**Figure 8:**
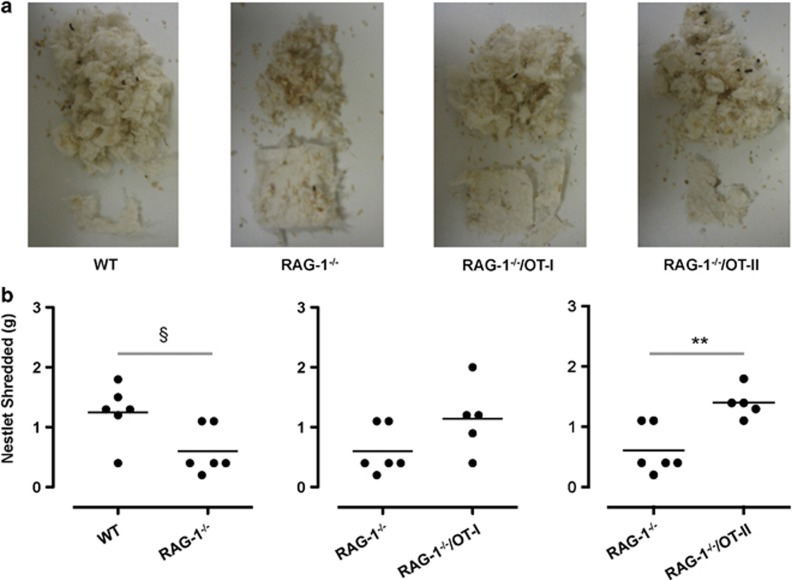
CD4^+^ but not CD8^+^ T cells revert the impaired nest construction of *RAG-1^−/−^* mice. (**a**) Representative pictures of the nestlet shredding activity of wild-type (WT), *RAG-1^−/−^*, *RAG-1^−/−^*/OT-I and *RAG-1^−/−^*/OT-II during an overnight test. (**b**) Quantitative analysis of nestlet shredding activity expressed as grams of nestlet shredded after an overnight test. Values are expressed as mean±s.e.m. of six mice and are representative of *n*=3–4 separate experiments. ^§^*P*<0.05 and **P<0.01 indicate significant values compared with WT C57BL/6 control and *RAG^−/−^* mice, respectively (Mann–Whitney *U*-test).

To explore the impact of T cells on other emotional behavior, we tested the three transgenic lines for their nesting activity, a standard test for measuring activities of daily living. Figure 8a shows representative pictures of the results obtained, whereas in Figure 8b, we report the quantitative results. Measurement of nestlet shredding showed a decreased ability of *RAG-1^−/−^* mice to perform this task compared with WT mice (Figure 8b). Similar to previous analysis, this impaired behavior was significantly reverted in *RAG-1^−/−^*/OT-II but not *RAG-1^−/−^*/OT-I mice (Figure 8b). Comparable results were obtained scoring nest quality (Supplementary Figure 1).

### No differences in systemic or gross brain structure between *RAG-1^−/−^*, *RAG-1^–/–^*/OT-I and *RAG-1^–/–^*/OT-II mice

**Figure 9:**
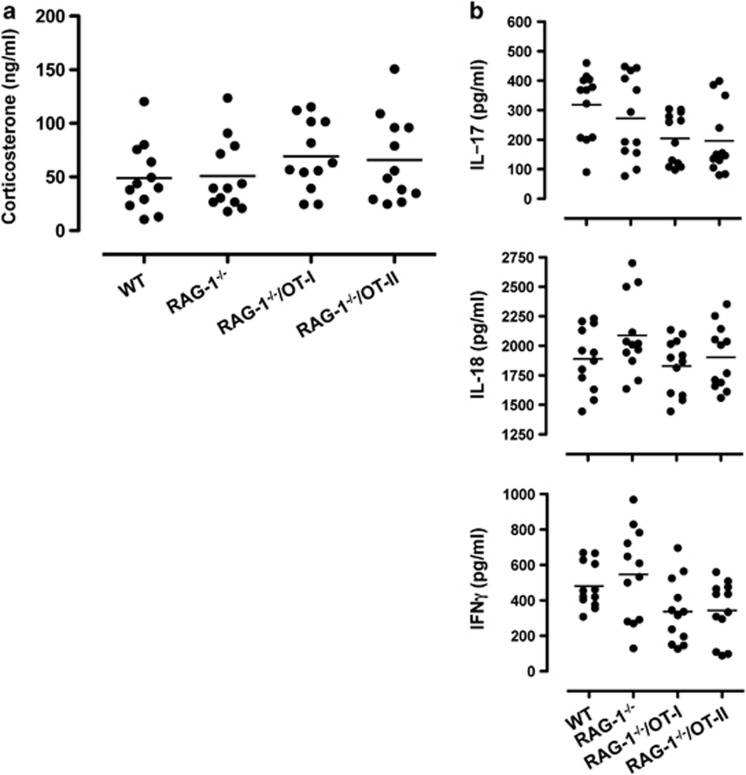
No differences in corticosterone or inflammatory cytokine serum levels between *RAG-1^−/−^*, *RAG-1^–/–^*/OT-I and *RAG-1^–/–^*/OT-II mice. Levels of corticosterone (**a**) or interleukin (IL)-17, IL-18 and interferon (IFN)-γ (**b**) in the serum of wild-type (WT), *RAG-1^−/−^*, *RAG-1^−/−^*/OT-I and *RAG-1^−/−^*/OT-II. Values are expressed as ng ml^−1^ or as pg ml^−1^ and are cumulative of *n*=2–3 experiments.

We next investigated whether the behavioral changes observed were due to changes in known behavioral modulating factors such as corticosterone. As shown in Figure 9a, there were no significant differences in the levels of circulating corticosterone between WT and *RAG-1^−/−^* mice or between *RAG-1^−/−^* and *RAG-1^−/−^*/OT-I and *RAG-1^−/−^*/OT-II mice.

Cytokines can induce behavioral changes (also known as sickness behavior^28^), a consequence of their modulatory effects on brain function. When we scanned the same samples for classical Th1, Th2, Th17 and ThGM-CSF cytokines, only interleukin (IL)-17, IL-18 and interferon-γ could be detected. However, none of these mediators was differentially modulated in *RAG-1^−/−^* and *RAG-1^−/−^*/OT-I and *RAG-1^−/−^*/OT-II mice compared with WT mice (Figure 9b), excluding the possibility that cytokines released by T cells or a latent state of infection as being responsible for the behavioral changes observed.

**Figure 10:**
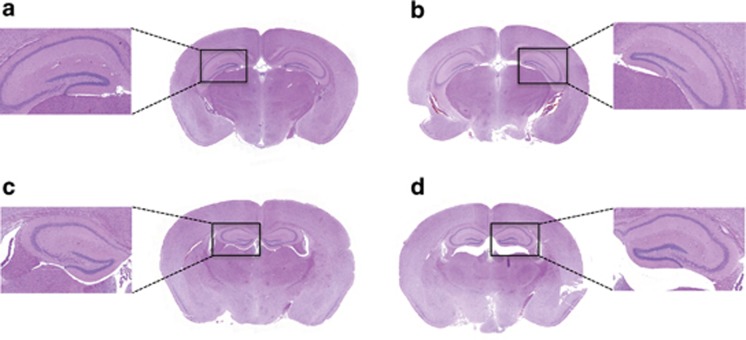
No differences in gross brain structure between *RAG-1^−/−^*, *RAG-1^–/–^*/OT-I and *RAG-1^–/–^*/OT-II mice. The pictures show the coronal hematoxylin and eosin -stained sections of brain from wild-type (**a**), *RAG-1^−/−^* (**b**), *RAG-1^−/−^*/OT-I (**c**) and *RAG-1^−/−^*/OT-II (**d**). The higher magnification represents the hippocampal area. The figures are representative of *n*=3–4 mice.

Similarly, analysis of general brain morphology and architecture of WT, *RAG-1^−/−^*, *RAG-1^−/−^*/OT-I and *RAG-1^−/−^*/OT-II mice showed comparable hematoxylin–eosin (Figures 10a–d, respectively) or luxol fast blue staining (data not shown) ruling out any contribution of infiltrated immune cells or general neuronal defect in the altered emotional behavior of tested mice.

### Brain gene fingerprint of *RAG-1^−/−^* and *RAG-1^−/−^*/OT-II

**Figure 11:**
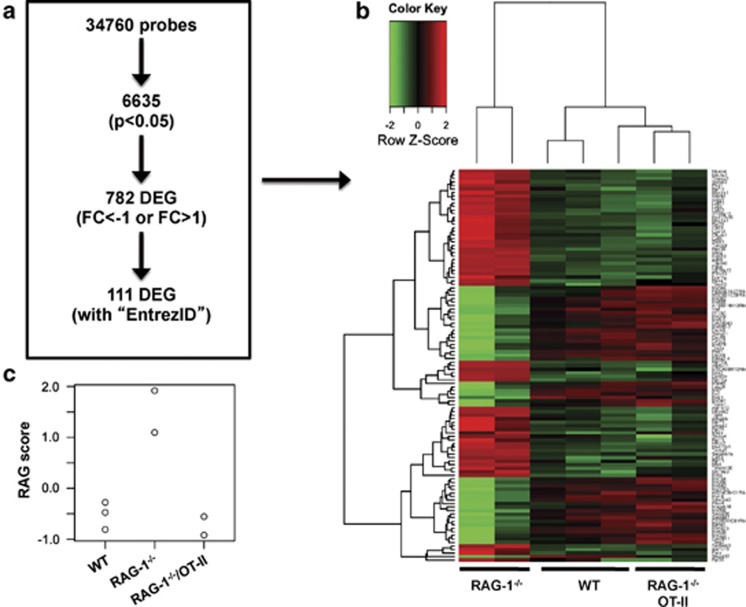
Heatmap and Canonical Correspondence Analysis on Microarray data of brain from wild-type (WT), *RAG-1^−/−^* and *RAG-1^–/–^*/OT-II mice. (**a**) Genes were filtered by a moderated t-statistics and fold change (FC). The heatmap analysis used annotated genes only (genes with EntrezID). (**b**) Hierarchical clustering and heatmap analysis of the filtered genes. *RAG-1^−/−^* samples showed a distinct cluster. (**c**) Similarity analysis for the features of *RAG-1^−/−^* in comparison with WT. Note that *RAG-1^−/−^*/OT-II mice had low scores and were equivalent to WT mice.

To unveil cellular and molecular mechanisms potentially responsible for the observed changes in emotional behavior, we took an unbiased approach and compared whole brain gene fingerprint of WT, *RAG-1^−/−^*, *RAG-1^−/−^*/OT-II mice. The flowchart in Figure 11a summarizes the results of this analysis. From the 34 760 probes present in the chip, 6635 were significantly modulated (*P*<0.05). This corresponded to 782 differentially expressed genes with a fold change (FC) value <−1 or >1, and 111 of them were annotated genes (genes with Entrez ID.)

Hierarchical clustering and heatmap analysis of these selected 111 genes and brain samples (Figure 11b and Table 1) showed that *RAG-1^−/−^* mice showed a distinct pattern of gene expression compared with *RAG-1^−/−^*/OT-II and WT mice. This result also suggested that *RAG-1^−/−^*/OT-II was similar to WT at the transcriptomic level. To further determine whether and how much *RAG-1^−/−^*/OT-II was more similar to WT than *RAG-1^−/−^*, we analyzed the similarities between the samples based on the gene expression pattern, focusing on the difference between WT and *RAG-1^−/−^*, which is our major interest in this study (see Materials and methods). The similarity analysis using these 111 genes showed that *RAG-1^−/−^*/OT-II was far more similar to WT than *RAG-1^−/−^* (Figure 11c). Importantly, the result of this similarity analysis was very similar using all the differentially expressed genes (data not shown), indicating that the result is robust and not dependent on the selection of genes by FC and annotation. These results were compatible with those of the behavioral analysis.

Pathway analysis provided further clues on the main differences between WT and *RAG-1^−/−^*. Using a third-generation pathway analysis approach,^29^ nine pathways were identified as been significantly modulated in *RAG-1^−/−^* compared with WT: two being activated (Parkinson's disease and RNA transport) and seven inhibited (Huntington's diseases, Alzheimer's disease, extracellular matrix–receptor interaction, olfactory transduction, focal adhesion, calcium signaling and small-cell lung cancer; summarized in Table 2 and reported singularly in Supplementary Figures 2–10).

## Discussion

The idea that a balanced mental state is a directly associated with general well-being can be traced back to the time of Decimus Iunius Iuvenalis. He was the first to state that a healthy mind is found in a healthy body (‘*mens sana in corpore sano*'). However, evidences gathered in our modern time suggests also the reverse, that is, that a *corpore sano*, and in particular a healthy immune system, might contribute to our mental well-being. In this study, we sought to provide direct experimental evidence of this hypothesis investigating first the emotional behavior of mice genetically void of T and B cells (the *RAG-1^−/−^* mice) and secondly assessing the specific contribution of CD4^+^ or CD8^+^ T cells.

Our results show a significant increase in anxiety-like behavior in *RAG-1^−/−^* mice as evaluated by the number of center entries in the open field test as well as the increased digging and marble-burying activities. Most interestingly, we also observed that CD4^+^ but not CD8^+^ T cells are able to revert significantly the exaggerated emotional response of *RAG-1^−/−^* mice. These results were not due to T-cell activation upon exposure of the mice to the behavioral paradigms (Supplementary Figure 11). The notion that CD4^+^ T cells has a preferential role as ‘mood stabilizer' compared with CD8^+^ T cells has long been suggested. Early studies on adult patients suffering from anxiety and obsessive-compulsive disorders have demonstrated immunological alterations including a significant increase of CD8^+^ and decrease of CD4^+^ lymphocytes compared with the healthy control group.^30^ The same abnormality has been observed in patients with autism, a disorder characterized also by obsessive-compulsive symptoms and anxiety disorders.^31^ A recent study has also demonstrated in a quantitative manner an inverse relationship between CD4 count and hospital-associated anxiety and depression.^32^ Finally, in one of his recent review AH Miller^33^ provided a comprehensive account of the multiple links between CD4^+^ T cells and depression, highlighting the importance of trafficking of T cells to the brain following stress as a way to reduce stress-induced anxiety-like behavior.^33^ However, a direct functional association between T cells and altered emotional behavior is still missing.

One of the major drawbacks in the life of patients suffering from anxiety-like behavior and/or obsessive-compulsive disorders is their inability to perform normal daily activities. Insightful epidemiological studies suggest that this ‘inability to cope' might be due to an emotional rather than a cognitive impairment.^34^ Our results are in line with this hypothesis and show a significant reduction in self-caring of *RAG-1^−/−^* mice compared with WT mice, as evaluated by the nesting test. Although these results suggest a link between self-neglect, anxiety-like behavior and immune suppression, more studies are needed to corroborate this hypothesis. These could include burrowing and hoarding, two other methods to test and quantify experimentally daily living activities.^35^

Nestlet shredding together with marble burying is also a reliable experimental model for obsessive-compulsive disorders and anxiety.^36^ We could not observe a significant difference in nestlet shredding after 60 min (data not shown), whereas we found a dramatic reduction in nest construction after an overnight test. We are tempted to explain these findings with the fact that, contrary to innate cells, adaptive immune cells are more involved in chronic disease and hence they usually exert their functions over a long period of time. In a similar way, one might speculate that immunosuppressed patients might present more difficulties in coping with long-term illness while being able to face problems as they came in.

Textbook immunology reports CD4^+^ T cells as ‘helper' cells because of their ability to modulate other cell functions, and these ‘helping' properties may go beyond antigen-presenting cell-mediated immunity. Groundbreaking studies by Kipnis and Schwartz^37, 38, 39^ has put CD4^+^ T cells at the center stage of neuroimmunology.^2^ Repopulation of *scid* mice with T cells from WT donors has been shown to improve significantly the impaired cognitive functions of these mice.^40^ Most interestingly, circulating and patrolling CD4^+^ T cells have been reported to convey constantly protective signaling to the brain, thus contributing to what is now known as ‘protective autoimmunity'.^41, 42, 43, 44^ In light of this concept, antigen-specific T cells, like myelin basic protein T-cell clones,^45^ circulate through the brain and sustain key neuronal processes and function such as neurogenesis, cognition and memory.^39^ Considering that the OT-II TCR transgene recognizes a non-endogenous antigenic peptide like OVA_323–339_, it is tempting to speculate that, at difference from cognition and memory, emotional behavior might require a less-stringent condition, that is, the simple presence of circulating CD4^+^ T cells. Indeed, their circulation through the brain or meningeal spaces, as suggested by Derecki *et al.*,^46^ might be enough to restore the emotional impairment that flares in immunocompromised conditions.^46^

Previous studies assessing the role of T cells on brain functions have used immunodepleting antibodies or cellular replacement in immunodeficient host like the *scid* mice.^40^ When we adopted similar approaches in our settings, we could not find any significant differences. Neither the depletion of T cells in C57/BL6 mice using anti-CD4 or anti-CD8 antibodies nor the repopulation of *RAG-1^−/−^* with purified CD4 or CD8 T cells had any effect on emotional behavior. The reason behind this apparent discrepancy might lay in the nature of the immunodeficient state that has been studied. In the *RAG-1^−/−^* mice, we have investigated how the absence of immune cells from prenatal development to adulthood influences neuronal networks and emotional behavior, whereas the immunodepletion or repopulation experiments refer to transient conditions (up to 2 weeks in our tests). This is quite an important aspect when one considers that neuropsychiatric disorders have often been linked to problems occurring at the developmental stage.^47, 48, 49^ Inflammation and infection at pre- and perinatal stages have been proved to be as powerful as maternal stress and trauma in causing long-term consequences on neuronal development and mental health.^50, 51, 52^ These clinical evidences hold true in experimental settings. Both peri- and prenatal administration of immunomodulatory agents such as TLR3 ligands and viral mimic polyinosine-polycytidylic, or TLR4 ligand and bacterial surrogate lipopolysaccharide, induced the development of schizophrenia- and autism-related behavioral changes including decreased exploratory activity and social interaction as adults.^53, 54, 55, 56, 57, 58^

Previous studies have shown that, like in pregnancy,^59^ CD4^+^ T cells with a skewed Th2 phenotype contribute to a controlled and trophic microenvironment in the brain upregulating neurotrophic factors such as glial-derived neurotrophic factor, brain-derived neurotrophic factor and insulin growth factor 1, or suppressing inflammatory mediators like tumor necrosis factor-α and IL-6.^46, 60^ Conversely, direct and indirect evidence have linked Th1 and Th17 cells to emotional disorders. T cells from individuals with generalized anxiety disorders show an enhanced capability to differentiate in Th17 cells.^61, 62^ Experimental evidences also suggest that Th17 cells preferentially accumulate in the brain of mice subjected to chronic restrain stress, whereas mice deficient in the RORγT (transcription factor necessary for Th17 differentiation) exhibited resistance to learned helplessness.^63^ T-bet (transcription factor necessary for Th1 differentiation) knockout mice show significantly reduced depressive-like behaviors provoked by repeated restraint stress.^64^ This is in line with clinical studies showing, for instance, the contribution of Th1 cytokines to the pathogenesis of neuropsychiatric manifestations of systemic lupus erythematosus.^65^ In light of these findings, we decided to investigate whether the absence or presence of circulating CD4^+^ T cells would impact neuronal gene development. Our microarray analysis provided us with a number of interesting findings.

A large number of micro-RNA(s) was found among the genes that were mostly downregulated. These gene expression modulators have been recently highlighted for their role in mental heath and are becoming increasingly popular in this field.^66^ Literature search for known targets of the micro-RNA we have identified provided us with few indications (Table 3). Further studies are needed to investigate the functions of these micro-RNA and their role in emotional behavior. Several quantitative polymerase chain reaction validated genes (data not shown) in our screening are known to control a variety of neuropsychological conditions. Synuclein-γ has been associated with Parkinson's disease^67^ and reported to be implicated in both cognitive and emotional functions.^68^ Von Willebrand factor has been shown to be significantly increased in schizophrenia,^69, 70, 71^ whereas changes in polycystic kidney disease 1 and tetratricopeptide repeat and ankyrin repeat containing 1) expression have been associated with bipolar disorders.^72, 73, 74^ S100a10, a recently suggested potential biomarker for suicide risk in mental disorders,^75^ was upregulated in *RAG-1^−/−^* and downregulated in *RAG-1^−/−^*/OT-II to WT level. Other interesting differentially expressed genes included ephrin type-B receptor 1 (*Ephb1*), whose genetic deletion in mice causes neuronal loss in the substantia nigra and spontaneous locomotor hyperactivity,^76^ myeloid/lymphoid or mixed-lineage leukemia 2 (*Mll2*), whose activity is required for memory formation,^77^ and Churchill domain containing protein 1 (*Churc1*), a neuronal development gene implicated with the occurrence of autism.^78^

Some of the implications of these changes in gene expression have been investigated with SPIA pathway analysis software. This showed inhibition of signaling pathways that control neurodegenerative disorders, such as Alzheimer's and Huntington's disease, in WT compared with *RAG-1^−/−^* mice further supporting the emerging view of these diseases as endowed with an autoimmune-related diseases component.^79^, ^80^, ^81^ Further differences were observed in a wide range of neuronal, sensory and basic cellular pathways that will be explored in future studies to detail the complex crosstalk between the neuronal and immune systems.

In conclusion, the results of this study shed new light on the complex crosstalk between the immune system and our emotional well-being, although future investigations are needed to corroborate our hypothesis. In fact, it would be interesting to explore the possible contribution of B cells to the emotional behavior of the *RAG-1^−/−^* mice as well as to confirm these results in mice expressing TCR transgene with different strength of signaling. Equally important, one might speculate the existence of CD4^+^ T-cell-specific factors that control emotional behaviors and their exploitation for the treatment of wide variety of mental disorders.

Beyond these experimental questions, the most important challenge for the future is to understand how T cells influence behavior and *vice versa*. The answer might lay in shared signaling pathway like *RAG* or the immune synapse: a signaling complex that has been named after the neuromuscular synapse and that allows the exchanges of information between antigen-presenting cells and T cells.^82, 83, 84^ Along these lines, recent studies have shown the existence of a subset of memory T cell in mice that produces acetylcholine in response to noradrenaline providing another way by which the immune system communicate with the nervous system.^85, 86^ Taken together, these findings might help the design of new therapies for mental health by restoring an impaired or absent immune system as observed in several autoimmune diseases.

## Figures and Tables

**Table 1:** Top-ranked genes (*n*=111) for the features of *RAG-1^−/−^* brain in comparison with wild-type

*Gene symbol*	*Probe ID*	*EntrezID*	RAG *score (gene score)*	*FC (*RAG*-WT)*	*FC (*RAG*-OTII)*	P*-value adj.*RAG *vs WT*	P*-value adj.*RAG *vs OTII*
*Mageb16*	1 054 5096	71 967	−2.040851531	−1.382	−2.412	0.080489	0.022479
*Vmn2r42*	1 055 9883	22 310	−1.93209053	−2.152	−1.793	0.021505	0.020203
*Mir300*	10 398 412	723 833	−1.88308172	−1.677	−2.085	0.017506	0.016937
*9530091C08Rik*	10 586 718	320 440	−1.794710154	−1.667	−2.619	0.104888	0.032618
*Phxr4*	10 583 203	18 689	−1.697861707	−1.052	−2.198	0.236372	0.040404
*Mir539*	10 398 418	723 917	−1.68592755	−1.867	−1.625	0.032518	0.037203
*A130014H13Rik*	10 402 560	319 630	−1.666278107	−1.767	−2.305	0.029595	0.018413
*Mir380*	10 398 388	723 859	−1.579352318	−1.198	−1.896	0.039846	0.018076
*Mir487b*	10 398 416	723 940	−1.515646393	−1.591	−1.606	0.029403	0.025115
*Gm9911*	10 578 017	10 010 1427	−1.495726048	−1.097	−1.857	0.171881	0.045496
*Tbrg3*	10 430 929	21 378	−1.480314578	−1.246	−1.955	0.059237	0.02181
*Mir323*	10 398 390	723 839	−1.459997834	−1.189	−1.849	0.084182	0.027581
*Mir680-2*	10 602 221	751 551	−1.436650249	−1.111	−2.109	0.136616	0.028476
*Mir665*	10 398 338	751 555	−1.426506241	−1.668	−1.866	0.0336	0.023984
*Mir376b*	10 398 408	723 934	−1.390698725	−1.236	−1.411	0.034845	0.023984
*Krtap2-4*	10 390 913	71 453	−1.383924657	−1.713	−2.176	0.049788	0.026248
*F830001A07Rik*	10 586 722	320 055	−1.360180695	−1.16	−1.984	0.170697	0.043923
*Mir382*	10 398 420	723 912	−1.358674308	−1.517	−1.887	0.029403	0.01938
*Mir154*	10 398 428	387 172	−1.302334846	−1.148	−1.479	0.022707	0.016937
*Gm10048*	10 529 953	625 026	−1.295861539	−1.173	−1.861	0.06009	0.02181
*Mir329*	10 398 392	723 842	−1.295645605	−1.373	−1.719	0.043559	0.024869
*Ephb1*	10 596 115	270 190	−1.28329288	−1.144	−1.534	0.044139	0.022479
*D230041D01Rik*	10 548 727	1 000 38615	−1.249725567	−1.103	−1.502	0.050443	0.023984
*Mir679*	10 398 396	751 539	−1.239666734	−1.275	−1.743	0.092767	0.038914
*Mir344*	10 564 235	723 931	−1.227043517	−1.01	−1.991	0.109837	0.023058
*4931406H21Rik*	10 413 216	77 592	−1.20459512	−1.236	−1.209	0.030727	0.027375
*Mir9-1*	10 493 191	387 133	−1.188744017	−1.132	−1.618	0.04865	0.02181
*Mir341*	10398350	723 846	−1.185006997	−1.323	−2.14	0.109543	0.032187
*Snord95*	10 375 501	1 002 16540	−1.140100065	−1.165	−1.229	0.049459	0.037703
*Tra2a*	10 544 638	101 214	−1.085592515	−1.154	−1.518	0.05765	0.027615
*Gm5887*	10 542 834	545 893	−1.068780678	−1.213	−1.068	0.023897	0.024869
*Snord37*	10 365 003	100 217 454	−1.052485206	−1.061	−0.627	0.017506	0.02181
*Mir543*	10 398 400	723 881	−1.046459099	−1.036	−1.118	0.021685	0.017462
*Myh9*	10 430 245	17 886	−0.951892542	−1.048	−0.884	0.021822	0.021872
*Il11ra1*	10 504 106	16 157	−0.935378718	−1.033	−1.167	0.02919	0.021613
*Vwf*	10 541 910	22 371	−0.913653403	−1.116	−1.213	0.024473	0.019653
*Malat1*	10 465 244	72 289	−0.881007189	−1.116	−1.756	0.136615	0.04157
*Celsr3*	10 589 130	107 934	−0.876228519	−1.171	−1.127	0.02257	0.019787
*Dock6*	10 591 614	319 899	−0.837490045	−1.295	−1.147	0.014287	0.016937
*Dock6*	10 591 630	319 899	−0.808120768	−1.142	−0.959	0.021097	0.018349
*Dock6*	10 591 612	319 899	−0.805918085	−1.083	−1.032	0.021505	0.017355
*Trank1*	10 589 761	320 429	−0.803294001	−1.008	−1.337	0.062753	0.029244
*Pkd1*	10 442 495	18 763	−0.798266404	−1.173	−1.073	0.020247	0.016937
*Snhg11*	10 478 073	319 317	−0.792852186	−1.038	−0.89	0.03061	0.03547
*Fn1*	10 355 403	14 268	−0.75577708	−1.188	−1.149	0.018698	0.016937
*Leng8*	10 549 615	232 798	−0.755496969	−1.053	−1.088	0.029574	0.024595
*Mll2*	10 432 298	381 022	−0.679798747	−1.088	−0.886	0.017506	0.016937
*Erdr1*	10 608 711	170 942	−0.516986696	−1.069	−0.546	0.019742	0.035677
*Mkks*	10 488 048	59 030	0.457287879	1.043	0.341	0.022707	0.18131
*Dynlt1c*	10 548 785	100 040 563	0.459997269	1.004	0.654	0.017506	0.01714
*Rpl10a*	10 355 173	19 896	0.485867583	1.117	0.793	0.021854	0.029315
*Nsa2*	10 411 363	59 050	0.552510543	1.192	0.814	0.018273	0.020521
*Atp6v1f*	10 536 895	66 144	0.573664963	1.027	1.039	0.021685	0.018076
*Rpsa*	10 385 034	16 785	0.573730493	1.054	0.675	0.017506	0.017355
*Plrg1*	10 492 757	53 317	0.578165545	1.006	0.899	0.017506	0.016937
*Hsd17b10*	10 602 592	15 108	0.594214684	1.012	0.98	0.021685	0.018382
*Snrpd2*	10 367 073	107 686	0.601279671	1.014	0.881	0.021505	0.019125
*Ly6c2*	10 429 573	100 041 546	0.616556062	1.066	0.885	0.024294	0.027767
*Bloc1s1*	10 373 594	14 533	0.639650101	1.016	1.1	0.021993	0.017462
*Rpp38*	10 479 749	227 522	0.640345029	1.128	0.834	0.017506	0.018347
*Wdr61*	10 593 740	66 317	0.668426554	1.012	1.07	0.022823	0.01851
*Cd53*	10 501 063	12 508	0.668970684	1.139	0.627	0.021505	0.040581
*Tm4sf1*	10 498 273	17 112	0.675247588	1.016	0.827	0.017506	0.016937
*Rpl29*	10 505 090	19 944	0.686092788	1.1	1.086	0.030792	0.026878
*Adh5*	10 496 475	11 532	0.693727155	1.039	1.025	0.017802	0.016937
*Nkain4*	10 490 551	58 237	0.698189047	1.015	1.213	0.021505	0.016937
*Rpl11*	10 454 097	67 025	0.70163422	1.163	1.003	0.039409	0.046635
*Rpl11*	10 517 457	67 025	0.706880397	1.009	0.903	0.021685	0.020078
*Ndufa2*	10 458 386	17 991	0.706987075	1.075	1.157	0.017506	0.016937
*Trim13*	10 415 784	66 597	0.708945695	1.285	0.768	0.021505	0.034966
*Rpl10a*	10 443 360	19 896	0.711172011	1.402	1.194	0.021505	0.018349
*Tspan6*	10 606 609	56 496	0.71734053	1.097	1.001	0.017506	0.016937
*Slc39a12*	10 469 389	277 468	0.724078447	1.08	1.054	0.018698	0.016937
*Txndc9*	10 396 064	98 258	0.737153463	1.094	1.077	0.021505	0.017462
*Kcnj13*	10 356 403	100 040 591	0.746292444	1.237	1.062	0.017506	0.016937
*S100a10*	10 493 995	20 194	0.749982194	1.295	1.167	0.017506	0.016937
*Trappc1*	10 377 508	245 828	0.753853858	1.109	1.257	0.022707	0.017462
*Ly6c1*	10 429 568	17 067	0.755009369	1.168	1.228	0.032378	0.025501
*Slc19a3*	10 356 145	80 721	0.76123195	1.042	0.765	0.02554	0.037851
*Bloc1s1*	10 457 924	14 533	0.763087509	1.124	1.198	0.021505	0.016937
*Tmem100*	10 380 285	67 888	0.763927693	1.001	0.715	0.021589	0.026199
*P2ry13*	10 498 367	74 191	0.766062492	1.278	0.969	0.017506	0.017355
*Hddc2*	10 362 394	69 692	0.799560244	1.01	1.071	0.021505	0.016937
*Sncg*	10 418 921	20 618	0.812109509	1.236	1.33	0.011381	0.012815
*Churc1*	10 396 694	211 151	0.830334656	1.017	1.336	0.037868	0.021562
*Scrg1*	10 571 865	20 284	0.838580022	1.22	1.228	0.025882	0.022109
*Rpl29*	10 490 824	19 944	0.8434953	1.052	1.155	0.039564	0.028208
*Hsd11b1*	10 361 234	15 483	0.850759562	1.097	1.145	0.017506	0.016937
*Snrpd2*	10 498 595	107 686	0.851183191	1.075	1.138	0.026256	0.021562
*Pgcp*	10 423 556	54 381	0.868297012	1.256	1.28	0.017506	0.016937
*Tpmt*	10 409 021	22 017	0.88208296	1.027	1.412	0.021822	0.016937
*Cd63*	10 367 436	12 512	0.888313271	1.17	1.461	0.022707	0.016937
*5730469M10Rik*	10 419 082	70 564	0.895272141	1.268	1.91	0.03237	0.017355
*Ly86*	10 404 606	17 084	0.897001413	1.274	1.271	0.021685	0.018349
*Akr1b10*	10 537 157	67 861	0.902194098	1.175	1.305	0.021505	0.016937
*Lgals1*	10 425 161	16 852	0.912182029	1.268	1.398	0.021505	0.016937
*N6amt2*	10 420 385	68 043	0.91317463	1.241	1.256	0.021685	0.018347
*Arhgdib*	10 548 892	11 857	0.929045264	1.003	1.328	0.058924	0.027787
*Ly6a*	10 429 564	110 454	0.94058187	1.438	1.499	0.025555	0.021402
*Fcer1g*	10 360 070	14 127	0.963847748	1.2	1.306	0.055767	0.039132
*Akr1c18*	10 407 435	105 349	0.964325333	1.047	0.762	0.024294	0.036537
*Serpinb9*	10 404 429	20 723	0.966649984	1.023	0.968	0.017506	0.016937
*Rfc4*	10 438 690	106 344	0.99077814	1.162	1.23	0.020247	0.016937
*Serpinb1a*	10 408 557	66 222	1.057850202	1.247	1.558	0.017506	0.013705
*Gstk1*	10 537 712	76 263	1.150030127	1.592	1.697	0.021505	0.016937
*Rpl11*	1 050 2745	67 025	1.167088017	1.307	1.558	0.037379	0.023823
*Stxbp4*	1 038 9795	20 913	1.173500167	1.086	1.948	0.091593	0.023603
*Rpl11*	1 045 1301	67 025	1.243624445	1.26	1.699	0.053335	0.025053
*Ppia*	1 054 5337	268 373	1.52055618	1.462	1.698	0.034726	0.023458
*Mela*	1 058 2545	17 276	3.221879948	4.675	2.776	0.002256	0.016937
*Rpl36*	10 378 783	54 217	3.792674002	2.358	3.245	0.109543	0.045167

Abbreviations: FC, fold change; WT, wild type.

The table shows the identity and statistical values of the top-ranked genes that were used for heatmap analysis (Figure 9b) and calculating the similarity score (*RAG* score) for the *RAG-1^−/−^* phenotype compared with the wild-type one (Figure 9c). Genes are ordered according to their relative contributions to the *RAG* score (i.e. the association with *RAG-1^−/−^* or WT; positive values indicate association with *RAG-1^−/−^*, whereas negative ones indicate association with WT). See Figure 9a for how genes were selected.

Probe ID is affymetrix ID.

FC is logged value.

**Table 2:** Signaling pathway impact analysis of *RAG-1^−/−^* brain

*Name*	*ID*	*pSize*	*NDE*	*pNDE*	*tA*	*pPERT*	*pG*	*pGFdr*	*pGFWER*	*Status*	*KEGGLINK*
Parkinson's disease	5012	117	62	4.21E−19	4.097	0.2	3.78E−18	4.73E−16	4.73E−-16	Activated	http://www.genome.jp/dbget-bin/show_pathway? mmu05012+66725+22202+22195+57320+68943+13063+104130+17991+17992+17993+225887+226646+227197+230075+407785+54405+595136+66046+66108+66218+66495+67130+67184+67264+67273+68198+68349+69875+72900+66925+66945+66152+66576+66594+66694+67003+67530+110323+12858+12859+12865+12866+12868+12869+20463+66142+75483+11949+11950+11957+228033+28080+67126+67942+71679+11739+11740+22334+22335+140499+67128+214084
Huntington's disease	5016	172	77	9.54E−18	−0.372	0.797	3.07E−16	1.92E−14	3.84E−14	Inhibited	http://www.genome.jp/dbget-bin/show_pathway?mmu05016+104130+17991+17992+17993+225887+226646+227197+230075+407785+54405+595136+66046+66108+66218+66495+67130+67184+67264+67273+68198+68349+69875+72900+66925+66945+66152+66576+66594+66694+67003+67530+110323+12858+12859+12865+12866+12868+12869+20463+66142+75483+11949+11950+11957+228033+28080+67126+67942+71679+15194+15182+433759+12914+328572+20020+20022+231329+66420+67710+69241+69920+14810+14812+108071+18798+16438+11739+11740+22334+22335+13063+72504+21780+12757+381917+68922+12913
Alzheimer's disease	5010	164	70	5.83E−15	−1.6765	0.558	1.12E−13	4.66E−12	1.40E−11	Inhibited	http://www.genome.jp/dbget-bin/show_pathway?mmu05010+66340+11487+13063+104130+17991+17992+17993+225887+226646+227197+230075+407785+54405+595136+66046+66108+66218+66495+67130+67184+67264+67273+68198+68349+69875+72900+66925+66945+66152+66576+66594+66694+67003+67530+11949+11950+11957+228033+28080+67126+67942+71679+110323+12858+12859+12865+12866+12868+12869+20463+66142+75483+20192+16438+16439+18798+14810+14811+14812+12288+12289+15108+14102+18125+78943+11816+19059+16971+14433+234664
ECM–receptor interaction	4512	84	28	0.000175059	−13.7865	5.00E−06	1.91E−08	5.98E−07	2.39E−06	Inhibited	http://www.genome.jp/dbget-bin/show_pathway?mmu04512+16772+16774+16775+16776+16779+226519+23928+12814+12825+12826+12827+12830+12833+12835+12842+12843+245026+81877+22371+20750+14268+21825+15529+20971+15530+16400+19699+11603
RNA transport	3013	161	56	2.67E−08	0.7715	0.271	1.43E−07	3.57E−06	1.79E−05	Activated	http://www.genome.jp/dbget-bin/show_pathway?mmu03013+433702+68092+56698+53975+69731+237221+20901+66069+60365+192170+56215+56009+237082+71805+114671+408191+72124+69912+54563+77595+107939+227720+20610+22218+13627+97112+66235+68969+53356+54709+56347+68135+78655+70047+102614+117109+208366+227522+54364+66161+67053+69961+386612+66231+26905+67204+218693+13681+13682+218268+230861+217869+108067+13667+209354+69482
Small-cell lung cancer	5222	86	22	0.026137813	−20.07421825	5.00E−06	2.20E−06	4.59E−05	0.000275271	Inhibited	http://www.genome.jp/dbget-bin/show_pathway?mmu05222+16400+56469+218772+17187+19211+30955+13063+12826+12827+12830+14268+16772+16774+16775+16776+16779+226519+23928+11797+19225+12567+12571
Olfactory transduction	4740	989	40	1	−23.1386	5.00E−06	6.60E−05	0.001179114	0.008253795	Inhibited	http://www.genome.jp/dbget-bin/show_pathway?mmu04740+100038860+18345+18369+235256+257884+257939+258027+258228+258247+258266+258278+258286+258407+258421+258446+258482+258483+258502+258533+258541+258570+258620+258648+258656+258677+258683+258712+258743+258922+258972+259006+259103+259105+404335+404336+56015+56860+57272+333329+19092
Focal adhesion	4510	199	41	0.095748092	−20.21166302	0.001	0.000981781	0.01486948	0.122722602	Inhibited	http://www.genome.jp/dbget-bin/show_pathway?mmu04510+16400+192176+286940+67268+67938+19211+30955+21894+70549+12814+12825+12826+12827+12830+12833+12835+12842+12843+14268+16772+16774+16775+16776+16779+19699+20750+21825+22371+226519+23928+245026+81877+12389+12390+11797+12445+16001+18596+107746+109905+57257
Calcium signaling pathway	4020	179	47	0.000966948	−5.8395	0.109	0.001070603	0.01486948	0.133825321	Inhibited	http://www.genome.jp/dbget-bin/show_pathway?mmu04020+320404+432530+18125+22334+22335+110891+20541+19059+12494+13869+18596+14810+14811+18438+228139+18803+18802+16438+16439+102093+18679+18682+68961+20190+20191+20192+18798+12288+12289+12286+12287+12290+108071+11550+12669+21338+21390+243764+26361+21924+11739+11740+11515+12291+239556+58226+11941

Abbreviations: ECM, extracellular matrix; pG, global *P*-values, obtained by combining the pPERT and pNDE using Fisher's method; pGFdr, global *P*-values after fdf correction; pGFWER, global *P*-value adjusted by the Bonferroni's method; pNDE, *P*-value by the number of differentially expressed genes (classical test for the enrichment of genes in a certain pathway); pPERT, *P*-value by perturbation (calculated based on the amount of perturbation measured in each pathway).

Microarray data of *RAG-1^−/−^* and wild-type brains were analyzed by a moderate t-statistics and fold change, and subsequently analyzed for the pathway enrichment with a bootstrap technique using the Bioconductor package, *SPIA*. Significantly modulated pathways were selected by a global pathway significance *P*-value with considering false discovery rate (*P*<0.05), which combines the enrichment and perturbation *P*-values.

The analysis was perfomed by SPIA (Bioconductor package).

**Table 3:** miRNA modulated in *RAG-1^−/−^* brain and their relative targets.

*miRNA*	*Cited in*	*Known targets*
Mir539	Bao B *et al. J Nutr* 2010;	Holocarboxylase synthetase
	Haga CL and Phinney DG. *J Biol Chem* 2012	Twist-related protein 1, polycomb complex protein BMI-1
Mir380	Hu K *et al. BMC Neurosci* 2012; Matsumoto S *et al. Biochem Biophys Res Commun* 2012	Unknown
Mir487b	Xi S *et al. J Clin Invest* 2013	Polycomb protein SUZ12, polycomb complex protein BMI-1, protein Wnt-5a, Myc proto-oncogene protein, GTPase KRas
Mir323	Qiu S *et al. J Transl Med* 2013; Fenoglio C *et al. Int J Mol Sci* 2012	Unknown
Mir680-2	None	Unknown
Mir665	Si H *et al. J Cancer Res Clin Oncol* 2013	Unknown
Mir376b	Korkmaz G *et al. Autophagy* 2012	Cysteine protease ATG4C and beclin-1
Mir382	Kriegel AJ *et al. Physiol Genom* 2012	Kallikrein 5
	Haga CL and Phinney DG. *J Biol Chem* 2012	Twist-related protein 1, polycomb complex protein BMI-1
Mir154	Milosevic J *et al. Am J Respir Cell Mol Biol* 2012	WNT/β-catenin pathway
Mir329	Khudayberdiev S *et al. Commun Integr Biol* 2009; Qiu S *et al. J Transl Med* 2013	Unknown
Mir679	None	Unknown
Mir344	Qin L *et al. BMC Genom* 2010	WNT/β-catenin pathway
Mir9-2	Rodriguez-Otero P *et al. Br J Haematol* 2011	Fibroblast growth factor receptor 1 and cyclin-dependent kinase 6
Mir341	None	Unknown
Mir543	Haga CL and Phinney DG. *J Biol Chem* 2012	Twist-related protein 1, polycomb complex protein BMI-1

Abbreviations: miR, micro-RNA. The table shows a list of miRNAs modulated in *RAG-1^−/−^* compared to *RAG-1^−/−^*/OT-II and wild type brains and the relative studies describing their molecular target(s).
