# Supplementary material for: CD4+ but not CD8+ T cells revert the impaired emotional behavior of immunocompromised RAG-1-deficient mice
Source: Transl Psychiatry. 2013 Jul 9;3(7):e280–. doi: 10.1038/tp.2013.54 (PMC3731786; doi:10.1038/tp.2013.54)
Supplement: Supplementary Figure Legends [file tp201354x12.doc]

**FIGURE LEGENDS**

**Supplementary Figure 1. CD4^+^ but not CD8^+^ T cells revert the impaired nest construction of RAG-1^-/-^ mice.** The bar graphs show the nest quality score assessed in an overnight test. The values are expressed as mean ± S.E.M. of 6 mice and are representative of n=3-4 separate experiments. ^§^ (*p* < 0.05) and ** (*p* < 0.01) indicate significant values compared to wild-type C57BL/6 control and RAG^-/-^ mice, respectively (Mann–Whitney U test).

**Supplementary Figure 2-10. Signaling pathway impact analysis of RAG-1^-/-^ and wild-type brains.** We have employed the bioconductor package *SPIA* that analyses both p-value and fold change and identifies significantly modulated pathways using Kyoto Encyclopedia of Genes and Genomes (KEGG). Nine pathways were significantly modulated between these two genotypes. Red coloured genes were differentially expressed between the groups (either upregulated or downregulated). **Supplementary Figure 2**. RNA transport (KEGG-ID, map03013); **Supplementary Figure 3**. Calcium signaling pathway (map04020); **Supplementary Figure 4**. Focal adhesion (map04510); **Supplementary Figure 5**. ECM-receptor interaction (map04512); **Supplementary Figure 6**. Olfactory transduction (map04740); **Supplementary Figure 7**. Alzheimer’s disease (map05010); **Supplementary Figure 8**. Parkinson’s disease (map05012); **Supplementary Figure 9**. Huntington’s disease (map05016); **Supplementary Figure 10**. Small cell lung cancer (map05222).

**Supplementary Figure 11. T cells are not activated in response to the behavioral paradigms.** Dot plots showing the CD3^+^-gated T cell population in wild-type mice tested (+ behavioral paradigm) or not (- behavioral paradigm) in the open field (**A**) or marble burying (**B**) tests. The hystograms show the comparison of their relative level of expression of CD69 (early T cell activation marker). Data are representative of n=3-4 separate experiments.
